# What is the new normal?

**DOI:** 10.4102/safp.v62i1.5235

**Published:** 2020-12-10

**Authors:** Robert Mash

**Affiliations:** 1 Division of Family Medicine and Primary Care, Faculty of Medicine and Health Sciences, Stellenbosch University, Cape Town, South Africa

The crisis arising from the coronavirus disease 2019 (COVID-19), has revealed the strengths and weaknesses of our health system and has led to opportunities for innovation. Many of these innovations will become part of our ‘new normal’ as we go forward. For example, web-meetings, home delivery of medication by community health workers,^1^ telephonic consultations or people visiting hospitals via video links.^2^ Our ability for adaptive leadership has been tested, as practitioners and practices were forced to continually reorganise and solve service-delivery problems.^3^

Interestingly, a global study by the World Organisation of Family Doctors did not show a relationship between the strength of primary care systems in different countries and the impact of the epidemic.^4^ Maybe the public health interventions (e.g. lockdown, case investigation, contact tracing, community screening and testing, isolation and quarantine) were more proactive in containing or suppressing the epidemic, whilst primary care was more reactive. The integration of public health and primary care responses may also have been an important factor, particularly within a community-oriented primary care (COPC) approach.^5^

Systems that had implemented key components of this COPC approach were able to build on these, such as teams of community health workers serving delineated geographical areas, and the functional integration of facility-based and community-based primary health care providers.^1^ The key deficiencies in implementation of COPC were equally obvious, such as poor community engagement and participation in constructing feasible responses, as well as the lack of inter-sectoral collaboration, particularly that of social services.^6^ This may relate to community resistance to restrictions imposed from outside and to the difficulties faced in acquiring food and other humanitarian assistance. Other commentators have made a link between the commitment of politicians and society to social solidarity or cohesion and the impact of the epidemic.^7^

Many of the issues that were put on hold during the crisis are still important to the ‘new normal’. The development of a new human resources for health policy in South Africa is vital, and its implications on family medicine. A recent survey amongst family medicine practitioners recommended that training programmes need to triple their output over the next 10-years to meet the modest goal of a family physician for each district hospital, health centre and sub-district.^8^

National Health Insurance (NHI) remains on the cards, and I have published a series of articles in the *South African Family Practice Journal* exploring the implications for family medicine practitioners.^9^ Dr Nicholas Crisp, a consultant on NHI to the National Department of Health, has asked for a series of webinars with members of the *SA Academy of Family Physicians* to hold discussions and help shape their plans. How NHI will work for private practice particularly is an issue he is keen to explore.

One of the key concerns in the primary health care response to COVID-19 has been to maintain services for other conditions and not to lose ground in the fight against HIV, TB, diabetes and other diseases. The *SA Academy of Family Physicians* in collaboration with AOSIS (Pty) Ltd eCPD^®^ (https://healthcare-ecpd.co.za/) has been instrumental in developing specialised web-based short courses on key issues. The first two courses have been launched on *Hypertensive Disorders in Pregnancy* and *Brief Behaviour Change Counselling*.

Hypertension in pregnancy has become one of the most important contributors to maternal and neonatal mortality, and new guidelines were published at the end of 2019.^10^ The short course presented by Dr Bob Patterson aims to ensure that we are all up-to-date with the new recommendations. Brief behaviour change counselling is a key competency for all primary care providers and applies across multiple behaviours such as tobacco-smoking, physical activity, healthy eating, alcohol consumption, adherence to medication and safe sex.^11^ The short course is presented by Dr Zelra Malan and includes feedback on the performance of such counselling in practice.
